# Oral calcium and vitamin D supplements differentially alter exploratory, anxiety-like behaviors and memory in male rats

**DOI:** 10.1371/journal.pone.0290106

**Published:** 2023-08-11

**Authors:** Sarawut Lapmanee, Sakkarin Bhubhanil, Siriwan Sriwong, Chaowalit Yuajit, Prapimpun Wongchitrat, Jarinthorn Teerapornpuntakit, Panan Suntornsaratoon, Jantarima Charoenphandhu, Narattaphol Charoenphandhu

**Affiliations:** 1 Department of Basic Medical Sciences, Faculty of Medicine, Siam University, Bangkok, Thailand; 2 Laboratory Animal Center, Thammasat University, Pathum Thani, Thailand; 3 College of Medicine and Public Health, Ubon Ratchathani University, Ubon Ratchathani, Thailand; 4 Faculty of Medical Technology, Center for Research and Innovation, Mahidol University, Nakon Pathom, Thailand; 5 Faculty of Medical Science, Department of Physiology, Naresuan University, Phitsanulok, Thailand; 6 Center of Calcium and Bone Research (COCAB), Faculty of Science, Mahidol University, Bangkok, Thailand; 7 Department of Physiology, Faculty of Science, Mahidol University, Bangkok, Thailand; 8 Physiology Division, Preclinical Sciences, Faculty of Medicine, Thammasat University, Pathum Thani, Thailand; 9 Institute of Molecular Biosciences, Mahidol University, Nakhon Pathom, Thailand; 10 The Academy of Science, The Royal Society of Thailand, Bangkok, Thailand; Poznan University of Life Sciences: Uniwersytet Przyrodniczy w Poznaniu, POLAND

## Abstract

Oral calcium and calcium plus vitamin D supplements are commonly prescribed to several groups of patients, e.g., osteoporosis, fracture, and calcium deficiency. Adequate and steady extracellular calcium levels are essential for neuronal activity, whereas certain forms of calcium supplement (e.g., CaCO_3_) probably interfere with memory function. However, it was unclear whether a long-term use of ionized calcium (calcium chloride in drinking water *ad libitum*), vitamin D supplement (oral gavage) or the combination of both affected anxiety and memory, the latter of which was probably dependent on the hippocampal neurogenesis. Here, we aimed to determine the effects of calcium and/or vitamin D supplement on the anxiety- and memory-related behaviors and the expression of doublecortin (DCX), an indirect proxy indicator of hippocampal neurogenesis. Eight-week-old male Wistar rats were divided into 4 groups, i.e., control, calcium chloride-, 400 UI/kg vitamin D_3_-, and calcium chloride plus vitamin D-treated groups. After 4 weeks of treatment, anxiety-, exploration- and recognition memory-related behaviors were evaluated by elevated pulse-maze (EPM), open field test (OFT), and novel object recognition (NOR), respectively. The hippocampi were investigated for the expression of DCX protein by Western blot analysis. We found that oral calcium supplement increased exploratory behavior as evaluated by OFT and the recognition index in NOR test without any effect on anxiety behavior in EPM. On the other hand, vitamin D supplement was found to reduce anxiety-like behaviors. Significant upregulation of DCX protein expression was observed in the hippocampus of both calcium- and vitamin D-treated rats, suggesting their positive effects on neurogenesis. In conclusion, oral calcium and vitamin D supplements positively affected exploratory, anxiety-like behaviors and/or memory in male rats. Thus, they potentially benefit on mood and memory in osteoporotic patients beyond bone metabolism.

## Introduction

Calcium and vitamin D are not only important for bone metabolism but also for certain neural functions, e.g., action potential, neuroplasticity, learning, memory and neuroemotional changes [1–3]. Since inadequate calcium and/or vitamin D intake can increase risk of osteoporosis and fracture, calcium and vitamin D supplements are widely used in osteoporotic patients [4–6]. Dietary calcium supplementation has been reported to increase bone mass and strength in both human [4] and mice [7]. After being absorbed into the body, vitamin D_3_ is normally converted to hydroxyvitamin D_3_ [25(OH)D_3_] and 1,25-dihydroxyvitamin D_3_ [1,25(OH)_2_D_3_], respectively, the latter of which is the active form for enhancement of intestinal calcium absorption (for review, please see [8]). Furthermore, vitamin D supplement is able to modulate energy balance, and the serum 25-hydroxyvitamin D_3_ [25(OH)D_3_] levels are linked to body composition [9]. For instance, an increase in 25(OH)D_3_ level by vitamin D_3_ supplementation was found to reduce body fat mass [10].

Regarding the effects of calcium and vitamin D on the neuroemotional changes, high serum and cerebrospinal fluid calcium levels were reported to decrease behavioral response and sleep duration [11]. Hypercalcemia due to primary hyperparathyroidism was reportedly associated with mania [12], whereas low serum vitamin D led to anxiety, depression, cognitive impairment, and structural changes in the animal brains [13,14]. Furthermore, an episode of calcium supplementation in drinking water (3 days to 2 weeks), which increased serum and brain calcium levels but decreased brain serotonin turnover, was associated with the enhanced learned helplessness and exploratory behaviors [15,16]. Vitamin D supplement could also alleviate some neurological symptoms in patients with Parkinson’s disease, and improve the depression-like behaviors in ovariectomized rats [17,18]. We thus hypothesized that oral calcium and/or vitamin D supplements with dosages known to keep balancing calcium status may have some beneficial effects on anxiety-like behavior and memory of male rats. It was noteworthy that inappropriate calcium supplementation was able to aggravate cognitive decline or dementia in certain groups of patients, e.g., female elderly with cerebrovascular disease [19].

The hippocampus is crucial for both anxiety response and memory in humans and rodents [20–22], and the hippocampal neurogenesis is often induced by factors that modulate learning and memory in rats. Moreover, a link between vitamin D and hippocampal neurogenesis via calcium signaling has been reported [23–25]. An increase in hippocampal neurogenesis was also found to alleviate anxiety- and depression-like behaviors in stressed mice [26]. Since oral calcium and/or vitamin D supplements may positively affect anxiety response and memory, they were further postulated to modulate the expression of doublecortin (DCX)—a neural system-specific microtubule-associated protein commonly used as an indirect biomarker of hippocampal neurogenesis and neuronal migration. The direct evidence that confirms the positive effects of extracellular calcium on neurogenesis is scant, but there are several purports suggesting that neurogenesis requires optimal levels of both extracellular and intracellular calcium [24,27].

**Fig 1:**
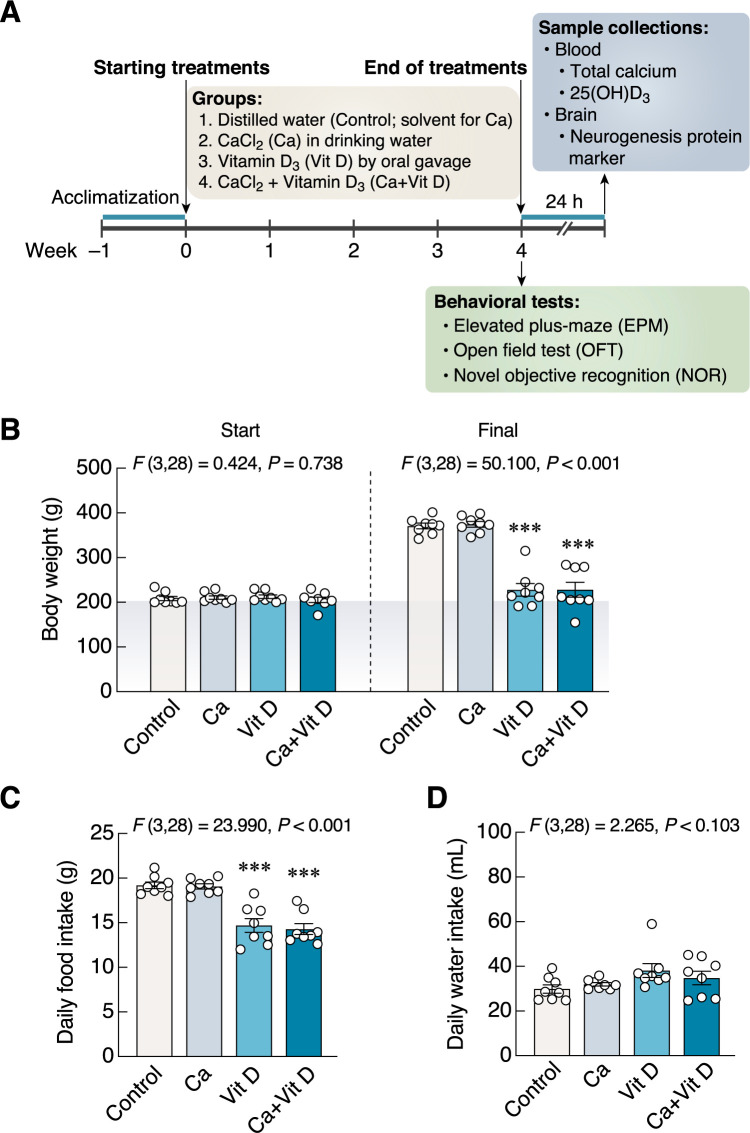
Experimental timeline, changes in body weight, daily food and water intakes. (A) Time course experiments. After 1-week acclimatization, all rats were assigned into 4 groups: control, calcium, vitamin D, and combined calcium and vitamin D supplements. Rats were treated with drinking water containing calcium chloride *ad libitum* and/or 400 UI/kg vitamin D_3_ and/or vehicle (soybean oil) orally via gavage tube for 4 weeks. At the end of treatment period, each rat was individually evaluated for anxiety levels, locomotor activity, and recognition memory by using elevated plus-maze (EPM), open field test (OFT), and novel object recognition (NOR), respectively. After behavioral tests (i.e., 24 h after the last supplementations), all rats were euthanized with isoflurane. (B–D) Changes in body weight, and food and water consumption in rats supplemented with calcium and/or vitamin D_3_ for 4 weeks. *P < 0.05, **P < 0.01, ***P < 0.001 vs. control. Ca, calcium supplement; Vit D, vitamin D supplement.

Therefore, the present study aimed to determine the effects of oral calcium supplement (calcium chloride dissolved in drinking water) with or without vitamin D supplement (dissolved in sterile soybean oil; given by gavage) on neurobehavioral changes (e.g., anxiety-like behaviors and object recognition) and the hippocampal DCX protein expression in male rats. The rats were divided into 4 groups accordingly, i.e., control (distilled water), calcium, vitamin D, and calcium + vitamin D (Fig 1A). We only used male rats in the present study in order to avoid variations and interferences owing to ovarian hormones, particularly estrogen-induced upregulation of calcium transporter expression (e.g., TRPV6) in the small intestine [28]. Since calcium/vitamin D supplement is often prescribed for aging patients with osteoporosis, our findings could be potentially translated for further optimization of calcium/vitamin D regimens to best benefit bone metabolism as well as mood and memory.

## Materials and methods

### Animals

Eight-week-old male Wistar rats weighing 180–200 g were obtained from Nomura Siam International Co., Ltd., Bangkok, Thailand. The rats were housed in a group of 2–3 rats/cage and acclimatized in controlled room conditions at temperature of 22 ± 1°C, humidity of 50 ± 5%, light intensity of 325 lux, and 12 h light/dark cycle. All rats were fed commercial rat chow (CP Co., Ltd., Bangkok, Thailand) and distilled water *ad libitum*. This study has been approved and carried out according to the Institutional Care and Use Committee of Thammasat University, Pathumthani, Thailand (Protocol number: 10/2561). Although certain bone diseases, e.g., osteoporosis, often affect females, we avoided using female rats in the present study because cyclical ovarian hormone release may interfere with anxiety-like behaviors and memory [29,30].

### Experimental design

After 1 week of acclimatization (Fig 1A), thirty-two rats were randomly divided into 4 groups of 8 rats each. Group 1 (Control) was given distilled water (solvent for calcium) *ad libitum* and sterile soybean oil (solvent for vitamin D_3_). Group 2 (Ca) was given calcium chloride in distilled water *ad libitum* plus soybean oil. Group 3 (Vit D) was given distilled water *ad libitum* and vitamin D_3_ dissolved in soybean oil. Group 4 (Ca + Vit D) was given both calcium chloride *ad libitum* plus vitamin D_3_ dissolved in soybean oil. In other words, the animals were fed distilled water (control) or calcium-enriched water in drinking bottles *ad libitum*, and were orally administered vitamin D_3_ or soybean oil via gavage tube. The last doses of supplements consisting of calcium and/or vitamin D_3_ were administered 24 h prior to conducting the last behavioral test (i.e., all behavioral tests were performed individually within 24 h after the last supplementation). Twenty-four hours after the end of supplementation, blood samples were collected by cardiac puncture for biochemical analyses [total calcium and 25(OH)D_3_ levels]. The timing of the individual behavioral tests was between 9:00 and 12:00 a.m., while the timing of the treatment was between 9:00 and 10:00 a.m.

The rats were finally euthanized with isoflurane anesthesia. After removal of whole brain, the hippocampus was collected for measurement of neurogenesis protein levels. DCX as a microtubule-associated phosphoprotein has been used as an indirect biomarker of newly born neurons and dendritic growth in adult dentate gyrus [31,32]. Regarding the DCX protein expression study, we carefully dissected the dorsal hippocampus, which contained the dentate gyrus, according to the modified methods of Vnek and Rothblat, and Heffner et al. [33,34]. Nevertheless, the brain samples may still contain some other hippocampal cells and fibers.

### Calcium/Vitamin D supplements

Provision of calcium and vitamin D_3_ was conducted for 4 weeks (Fig 1A). Rats were administered *ad libitum* with 15 mM calcium chloride solution (catalog no. 21114, Sigma Chemical Co., St. Louis, MO, USA.) and 12 mM monosaccharides (i.e., glucose; catalog no. 1009378, Ajax Finechem Pty Limited, New South Wales, Australia and galactose; catalog no. 0802339, Asia Pacific Specialty Chemicals Ltd., New South Wales, Australia) and 46.5 mM sodium chloride (catalog no. S5068-1-1000, Quality Reagent Chemical (Qrëc^TM^, New Zealand) according to the formula of Suntornsaratoon et al. (2015) [35]. Distilled water (solvent) or calcium chloride solution was added in drinking bottles for daily treatment.

Vitamin D_3_ (catalog no. C9756, Sigma Chemical) was dissolved in sterile soybean oil (Thanakorn Vegetable Oil Products Co., Ltd., Thailand) and given orally at 400 IU/kg body weight of rats (soybean oil volume of 0.3 mL), as previously reported by Vieth (2004) and Detregiachi et al. (2016) [36,37].

### Body weight, food intake and water intake measurements

Rats were daily weighed in grams. The starting and final body weights of rats were recorded, and changes in daily weight gain were calculated. Food and water intakes were calculated from weight/volume before and after 24 h in the metabolic cage (model 3700M071-01CS; Tecniplast, Varese, Italy).

### Elevated plus-maze test (EPM)

The maze was a black plastic open-topped box and elevated 50 cm from the floor. The EPM was composed of two open arms (50 × 10 cm) aligned at a right angle to the perpendicular closed arms (50 × 10 × 40 cm). Each rat was gently placed into the central square of the maze, then allowed to explore the EPM for 5 min. The time spent and number of entries into the open arms were recorded by an infrared camera. A lower value of anxiety index indicated anxiolysis [38–40]. Anxiety index was calculated according to the equation:

Anxiety index = [1–[(open arm time/total time) + (open arm entries/total entries)]]/2

### Open field test (OFT)

The open field apparatus was a black plastic box (76 cm long × 57 cm wide × 35 cm high) with grid floor divided into inner and outer zones. Each rat was gently placed in the corner squares and given 5 min to explore. Time spent in each zone of arena, total line crosses, rearing, and grooming were recorded by infrared video camera. Changes in number of the total line crosses and rearing represented exploration and locomotor activity [41,42].

### Novel object recognition (NOR)

NOR was performed in a black plastic box (63 cm long × 63 cm wide × 45 cm high) with a video camera suspended above in 360 lux room light. The procedure was performed according to Lapmanee et al., 2017 [42]. After a training session with familiar objects (ceramic bottles), the objects were changed to glass paperweight and ceramic peppermills. Object-exploring behaviors that ran for 3 min consisted of sniffing, licking and touching. A decrease in percent recognition index indicated cognitive impairment, which was calculated according to the equation:

$$\%Recognition\;index=\frac{\left[(novel\;object\;exploring\;time-familiar\;object\;exploring\;time)\right]}{Total\;exploration\;time}\times 100$$

### Serum total calcium and 25(OH)D_3_ analyses

Blood samples (5 mL/animal) were obtained from rats anesthetized with 5% isoflurane (Minrad Inc., New York, USA). Whole blood was placed at room temperature for 30 minutes. After centrifugation (3,000 rpm, 10 min, 4°C), serum samples were collected and kept at –80°C until measurement of total calcium levels by an automated biochemical analyzer (Dimension RxL, Dade Behring Co Ltd., Minnesota, USA) and 25(OH)D_3_ levels by immunoassay (catalog no. KAP1971; DIAsource ImmunoAssay, Louvain-La-Neuve, Belgium), respectively.

### Western blot analysis

Whole brain was removed from the skull and immediately frozen in liquid nitrogen for analysis of protein expression. The hippocampus was dissected following the methods of Heffner et al. (1980) [33]. The hippocampus tissue was mixed in lysis buffer with protease and phosphatase inhibitor cocktails (Sigma). Total protein concentration was determined by using BCA Protein Assay kit (Thermo Scientific Inc., Waltham, MA, USA). Protein samples (50 μg) were added into each well of 10% SDS-PAGE and transferred onto PVDF membranes, methanol soaked (Amersham Biosciences, New Jersey, USA). Thereafter, the membranes were incubated with 1:1,000 rabbit polyclonal anti-doublecortin antibody (catalog no. AB18723; Abcam, Cambridge, UK), or 1:2,000 rabbit polyclonal anti-β-actin antibody (catalog no; AB8227, Abcam) overnight at 4°C. After the washing step, membranes were incubated with 1:2,000 goat anti-rabbit IgG H&L (HRP) secondary antibody (catalog no. AB205718; Abcam) at room temperature for 2 h. Protein bands were visualized by using an enhanced chemiluminescence western blotting detection system (Luminata Crescendo Western HRP substrate; Merck‐Millipore, Darmstadt, Germany) on the X-ray film (Amersham Biosciences). Densitometry was determined by using ImageJ (National Institutes of Health, Bethesda, Maryland, USA). The DCX protein expression level relative to β-actin in the control group was normalized to 1.

### Immunofluorescent analysis of terminal deoxynucleotidyl transferase dUTP nick end labeling (TUNEL)

The TUNEL-positive cells represent apoptotic cells. Therefore, in order to assess apoptosis of cells in the dentate gyrus, TUNEL histochemistry was performed according to the manufacturer’s instruction using CF^®^ dye TUNEL assay apoptosis detection kit (catalog number CF488A; Biotium, CA, USA). Briefly, paraffin brain sections (5 μm in thickness) were deparaffinized and treated with DNaseI before TUNEL staining was set up as positive control. The sections were then mounted with mounting media containing 4′,6-diamidino-2-phenylindole (DAPI; Vector Laboratories, CA, USA) to stain nuclei, and examined by using Olympus BX51 microscope with fluorescence illuminator (×40 magnification; Olympus, Tokyo, Japan). All positive cells were quantified by counting all cells with fluorescent signals in each histological sample using ImageJ (i.e., counting all cells and nuclei in a section). The percentage of apoptotic cells was calculated from the number of TUNEL-positive cells (green color) and the total number of DAPI-stained nuclei (blue color), as follows.


$$TUNEL\;positive\;cells\;(\%)=\frac{TUNEL\;positive\;cells}{total\;number\;of\;nuclei\;}\times 100$$

### Statistical analyses

The data are expressed as means ± standard error of mean (SEM). The significance of the differences between groups was analyzed using one-way analysis of variance (ANOVA) with Dunnett’s multiple comparison test. The *F*-values, degree of freedom (df), and *P*-values were also presented. The level of significance was *P* < 0.05. Two-way ANOVA analysis was performed to determine the effects of two factors, i.e., calcium and vitamin D. All statistical tests and graphs were analyzed and plotted by using GraphPad Prism 7 (GraphPad Software Inc., San Diego, CA, USA).

## Results

### Calcium alone increased urinary calcium levels, while vitamin D_3_ decreased food intake and increased serum 25(OH)D_3_ levels

Baseline body weights were similar among the four experimental groups (*F*(3,28) = 0.424, *P* = 0.738) (Fig 1B). After 4 weeks of calcium and/or vitamin D supplementation, the final body weights of rats in the vitamin D and combined calcium and vitamin D groups significantly decreased (*F*(3,28) = 50.100, *P* < 0.001) (Fig 1B). Consistent with the final body weight, a decrease in daily food intake was observed in the vitamin D group as compared with the control group (*F*(3,28) = 23.990, *P* < 0.001) (Fig 1C). Despite showing no statistical significance, the vitamin D group had a tendency to increase daily water intake (*F*(3,28) = 2.265, *P* = 0.103) (Fig 1D). We did not observe any overt adverse reactions, e.g., fatigue, abnormal movement, immobility, infectious disease, bleeding, diarrhea, etc.

**Fig 2:**
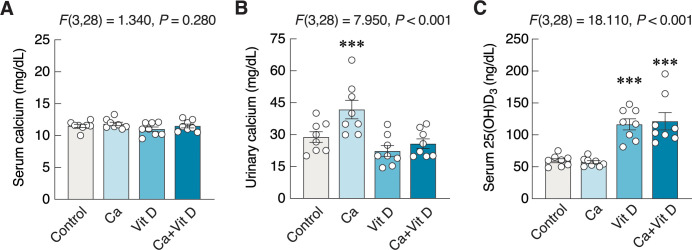
Changes in calcium and 25-hydroxyvitamin D_3_ [25(OH)D_3_] levels. (A) The serum calcium, (B) urinary calcium, and (C) serum 25(OH)D_3_ in rats supplemented with calcium and/or vitamin D_3_ for 4 weeks. ***P < 0.001 vs. control. Ca, calcium supplement; Vit D, vitamin D supplement.

Regarding analyses of urine and serum samples, there was no change in the total calcium level (*F*(3,28) = 1.340, *P* = 0.280) (Fig 2A), whereas a significant increase in urinary calcium level was observed in the calcium-treated group (*F*(3,28) = 7.950, *P* < 0.001) (Fig 2B). As expected, the two groups of rats that received vitamin D exhibited increases in serum 25(OH)D_3_ levels as compared to the control group (*F*(3,28) = 18.110, *P* < 0.001) (Fig 2C). However, there was no interaction among all parameters in the present study as analyzed by two-way ANOVA. All raw data are also provided as supplementary information.

### Vitamin D_3_ alone exhibited anxiolytic-like action

**Fig 3:**
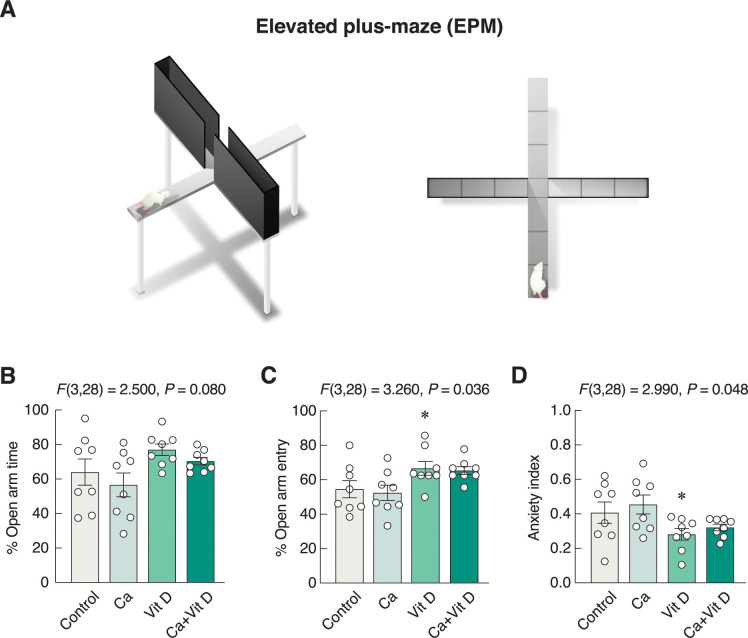
Changes in anxiety-related behaviors. (A) The anxiety-like behaviors were evaluated by EPM. (B) The percent open arm time, (C) the percent open arm entry, and (D) the anxiety index in rats supplemented with calcium and/or vitamin D_3_ for 4 weeks. *P < 0.05 vs. control. Ca, calcium supplement; Vit D, vitamin D supplement.

Anxiety-like behavior was determined by EPM as depicted in Fig 3A. The 4-week vitamin D_3_ supplement alone tended to increase time spent in the open arms (*F*(3,28) = 2.500, *P* = 0.080) (Fig 3B) and significantly increased open arm entry (*F*(3,28) = 3.260, *P* = 0.036) (Fig 3C). Thus, as shown in Fig 3D, the vitamin D-supplemented group exhibited a lower anxiety index than the control group (*F*(3,28) = 2.990, *P* = 0.048), suggesting that it had an anxiolytic-like action.

**Fig 4:**
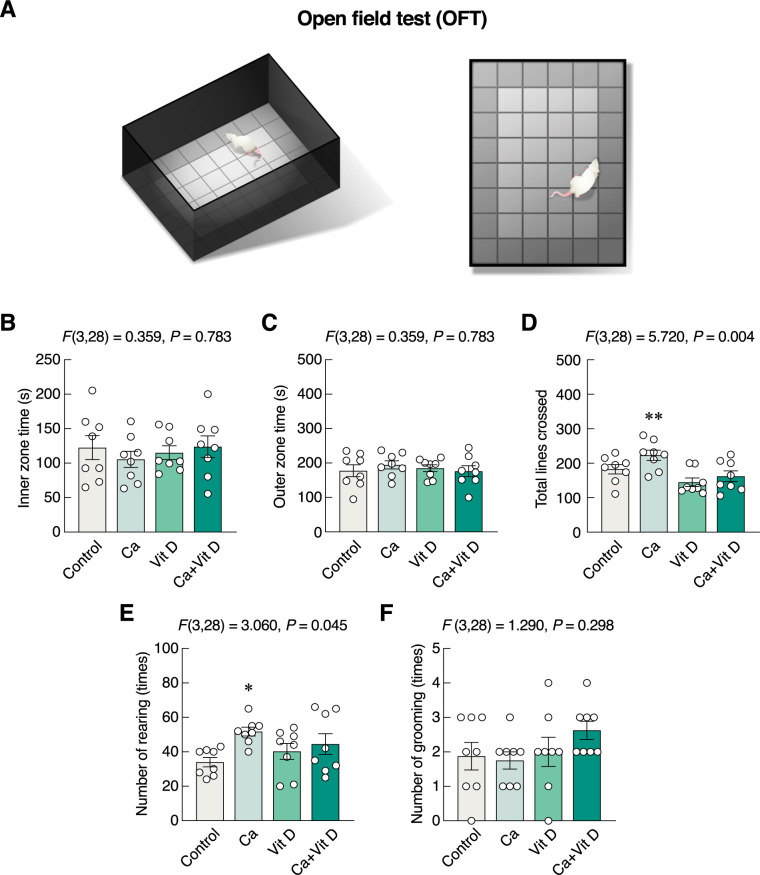
Changes in exploration and locomotor activity. (A) The behavioral profiles were evaluated by OFT. (B) The inner zone time, (C) outer zone time, (D) number of total line crossed, (E) number of rearing, and (F) number of grooming in rats supplemented with calcium and/or vitamin D_3_ for 4 weeks. *P < 0.05, **P < 0.01 vs. control. Ca, calcium supplement; Vit D, vitamin D supplement.

In OFT (Fig 4A), the numbers of line crossing and rearing were used as proxy indicators of locomotor activity. Although none of the supplementations altered time spent in the inner zone (*F*(3,28) = 0.359, *P* = 0.783) (Fig 4B), time spent in the outer zone (*F*(3,28) = 0.359, *P* = 0.783) (Fig 4C), or number of grooming (*F*(3,28) = 1.290, *P* = 0.293) (Fig 4F), calcium-supplemented group showed significant increases in the number of total lines crossed (*F*(3,28) = 5.720, *P* = 0.004) (Fig 4D) and number of rearing (Fig 4E), suggesting that calcium supplement alone was able to enhance locomotor activity.

### Calcium alone improved object recognition memory

**Fig 5:**
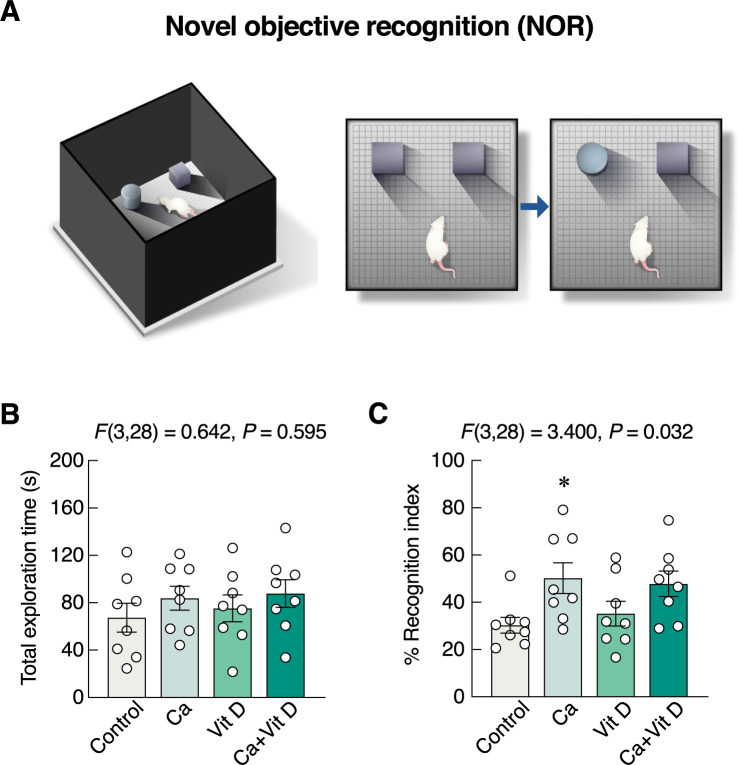
Changes in recognition memory. (A) The behavioral profiles were evaluated by NOR. (B) The total exploration time, and (C) percent recognition index in rats supplemented with calcium and/or vitamin D_3_ for 4 weeks. *P < 0.05 vs. control. Ca, calcium supplement; Vit D, vitamin D supplement.

NOR test was used to evaluate the differences between the novel and familiar object exploring times (Fig 5A). There were no significant differences in the total exploration time among the experimental groups (*F*(3,28) = 0.642, *P* = 0.595) (Fig 5B). However, the percent recognition index for the novel object in the calcium-supplemented group was higher than that in control group (*F*(3,28) = 3.400, *P* = 0.032) (Fig 5C). The aforementioned results suggested that calcium alone, but not vitamin D_3_, was capable of improving object recognition memory.

### Calcium and/or vitamin D_3_ upregulated hippocampal DCX protein expression

**Fig 6:**
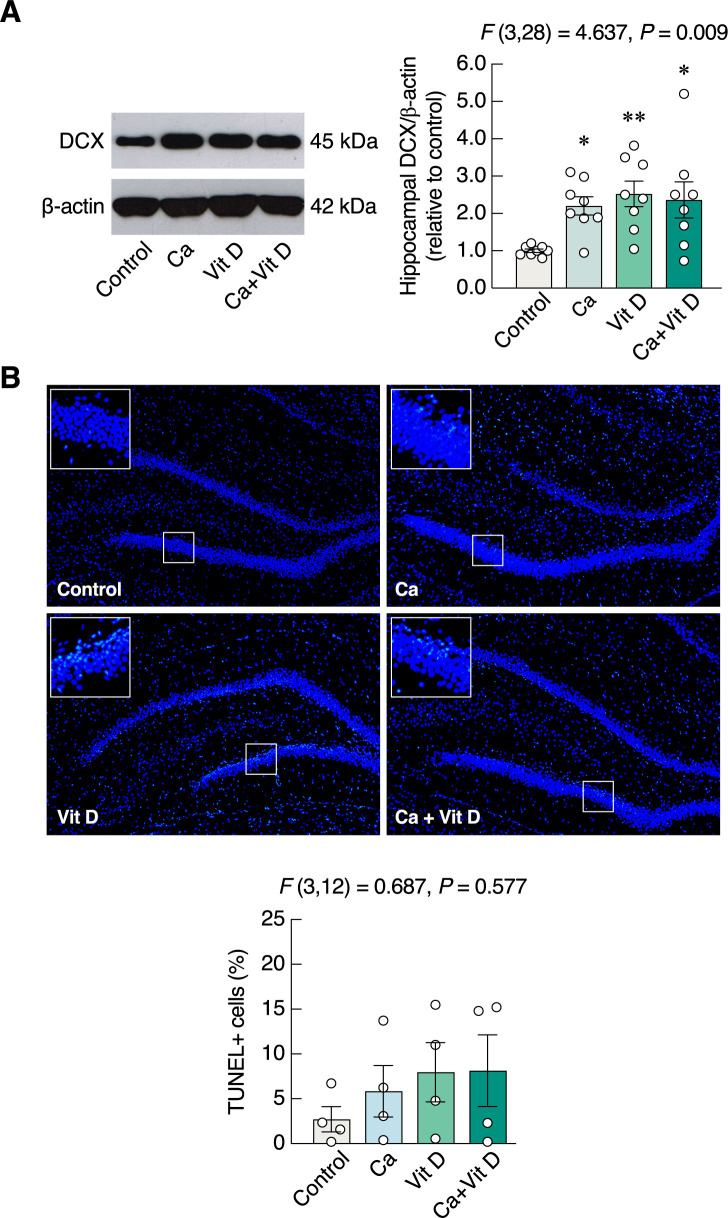
Expression of hippocampal doublecortin (DCX) and cell apoptosis in dentate gyrus. (A) Representative and relative protein expression levels of hippocampal doublecortin (DCX) and β-actin in rats supplemented with calcium and/or vitamin D_3_ for 4 weeks. (B) Representative fluorescent images (10×) of TUNEL-positive (green color) and nuclei counterstained with DAPI (blue color), and percentage of TUNEL-positive cells relative to total number of nuclei in dentate gyrus. *P < 0.05, **P < 0.01 vs. control. Ca, calcium supplement; Vit D, vitamin D supplement.

Since hippocampus plays a pivotal role in object recognition memory based on memory formation, the calcium/vitamin D supplements probably helped modulate the hippocampal neurogenesis. Hence, the proxy biomarker of adult neurogenesis—i.e., DCX protein—was determined by Western blotting analysis. We found that the hippocampal DCX protein levels of calcium-supplemented as well as vitamin D-supplemented groups were significantly upregulated (*F*(3,28) = 4.637, *P* = 0.009) (Fig 6A), as compared to the control group. DCX protein expression was also upregulated the calcium plus vitamin D group (Fig 6A). Meanwhile, calcium and/or vitamin D did not induce cell apoptosis in dentate gyrus as determined by TUNEL assay (*F*(3, 12) = 0.687, *P* = 0.577) (Fig 6B).

## Discussion

Our study has provided evidence that showed the positive effects of calcium and/or vitamin D_3_ on anxiety-like behaviors and object recognition memory. Calcium is one of the nutritional supplements recommended for postmenopausal women to alleviate bone loss and osteoporosis [4,5]. Under normal conditions, since serum calcium levels are tightly regulated and maintained within a narrow physiological range, it was not surprising to observe no change in serum calcium levels among the four groups in the present study. Although acute calcium administration and some calcium formulae might slightly increase serum calcium levels [43], an increase in serum calcium is often transient due to the fact that hypercalcemia is able to induce urinary calcium excretion as seen in Fig 2B. Thus, after 4-week calcium supplementation, the serum calcium levels were apparently normal. Nevertheless, some previous investigations have suggested that calcium intake within normal ranges did not induce hypercalciuria [44], but calcium intake in the present calcium-supplemented group was probably in a higher range, which might suppress parathyroid hormone response, thereby resulting in a reduction in renal calcium reabsorption and enhancing renal calcium excretion [45].

Regarding the behavioral responses, calcium supplementation was found to increase the exploratory behaviors and object recognition memory (NOR test) together with an increase in hippocampal DCX protein expression and no change in apoptosis in dentate gyrus. Our finding partially agreed with previous investigations showing that calcium supplement alone in drinking water was able to increase brain calcium levels (whole brain), which could contribute to high exploration activities [43] and central serotonin levels in rats [16]. The increased calcium levels in cerebral cortex, hippocampus, forebrain and brainstem could facilitate neurogenesis—a process known to increase memory capacity. The enhanced adult neurogenesis in dentate gyrus probably occurred via calcium influx through T-type calcium channels and calcium/calmodulin-dependent protein kinase II modulation [46,47]. Moreover, since there were crosstalks between parathyroid hormone-related protein (PTHrP)/brain calcium metabolism and adrenergic/serotonergic pathways, the underlying mechanisms of neurogenesis were probably related to norepinephrine and serotonin modulation that could increase the excitatory drive to the hippocampal circuit for neurogenesis [15,48–50]. Serotonin directly activated 5-HT3 receptors and also affected the function of PTHrP, which was capable of enhancing brain c-Fos expression and inducing a neuroprotective feedback loop through the L-type calcium channels [51,52]. Xu et al. also showed that a 5-HT3 receptor agonist (phenylbiguanide) was able to upregulate c-Fos expression in cortex and hippocampus [53]. Meanwhile, the binding of PTHrP to PTH/PTHrP receptor was capable of mediating neuroprotection against calcium-induced excitotoxicity [51,52]. Taken together, the present prolonged calcium supplementation might alter brain monoamine levels, thereby enhancing recognition memory and emotionality in rats, consistent with some previous human studies that showed a reduction in dementia risk by dietary calcium supplementation [54].

Apart from calcium supplementation, vitamin D_3_ is also crucial for the maintenance of bone structure, enhancement of intestinal calcium absorption [8] and brain functions [5,25]. Herein, there were significant increases in the levels of serum 25(OH)D_3_ in the vitamin D_3_-supplemented groups (Fig 2C). Under normal conditions, dietary vitamin D_3_ is transformed into 25(OH)D_3_ by CYP27A1 and CYP2R1 in several cells, particularly hepatocytes. However, during calcium imbalance with hypocalcemia, a conversion of 25(OH)D_3_ to 1,25(OH)_2_D_3_—the active form vitamin D_3_—is markedly enhanced by 1α-hydroxylase (CYP27B1) in the renal tubular cells. Although serum 1,25(OH)_2_D_3_ level was not determined in the present study, the absence of changes in serum and urinary calcium in the vitamin D-supplemented groups suggested that calcium homeostasis has already been kept in balance, and thus the levels of 1,25(OH)_2_D_3_ were probably unaltered [55].

The elevated 25(OH)D_3_ levels during vitamin D_3_ supplementation could lead to body fat mass reduction [10,37]. It was reported that vitamin D intake repressed fatty acid synthase activity in adipose tissue [56,57]. In addition, vitamin D was able to enhance lipid oxidation and energy consumption via agouti-related protein/neuropeptide Y [58,59]; therefore, there was a significant reduction in body weight in the groups receiving vitamin D_3_, but not calcium alone. Indeed, vitamin D_3_ is able to reduce body weight by a number of mechanisms. For example, it could modulate pancreatic insulin secretion and insulin sensitivity, thereby reducing food intake, white adipose tissue weight and body weight [59–61]. However, a decrease in body weight did not affect locomotor activity in the vitamin D_3_-supplemented rats, indicating that the present dosage of vitamin D_3_ supplement was not toxic. Evaluation of altered locomotor activities can be used to indicate toxicity, as reviewed by Gauvin et al. (2019) [62].

Furthermore, vitamin D_3_ supplement probably produced anxiolytic action, at least in part, by upregulating DCX protein expression, but had no effect on learning and memory. Normally, circulating 25(OH)D_3_ is capable of passing across the blood-brain barrier and can be converted into 1,25(OH)_2_D_3_ in glial cells and neurons [63], suggesting that changes in serum 25(OH)D_3_ levels eventually modulate neural circuits and brain functions. It was previously reported that the increased levels of serum 25(OH)D_3_ were correlated with a reduced risk of depression and anxiety disorder [64,65]. The positive findings of vitamin D and a reduction in anxiety were obtained from studies using various behavioral tests, e.g., OFT, the light-dark box, and the elevated plus-maze in the ovariectomized rats [66]. At the cellular level, vitamin D protected and enhanced growth of neuronal cells by inducing production of growth factors, e.g., nerve growth factor, glial cell line-derived neurotrophic factor, and neurotrophin 3 [67,68]. Moreover, it could also modulate biosynthesis of certain neurotransmitters—such as serotonin through tryptophan hydroxylase 2—and neurotrophic factors, thereby modulating mood and anxiety-like behaviors [69]. A number of previous investigations also reported the improvement of mood status, antioxidant and anti-inflammatory responses in vitamin D-treated type 2 diabetic patients [66,70]. Consistent with the behavioral studies, changes in the expression levels of essential molecules for learning and memory as well as neuronal proliferation, differentiation, survival and growth of hippocampal neurons—e.g., nerve growth factor and CREB—were detected [71,72]. In addition, the upregulation of neuronal biomarker of hippocampal neurogenesis could contribute to an alleviation of anxiety- and depression-like behaviors [26]. These data have supported our hypothesis that vitamin D_3_ supplementation exerted an anxiolytic-like action. Although its exact underlying mechanisms are unclear, hippocampal neurogenesis as indicated by DCX expression probably contributed to the process.

Nevertheless, the exact explanation regarding the absence of change in percent recognition index (Fig 5C) in Ca+Vit D is unclear. It was evident that calcium supplement alone was able to induce some positive effects on brain, e.g., increased exploration activities [15,43]. Although vitamin D often helps enhance the intestinal calcium absorption to increase body calcium, it also triggers a number of counterbalancing mechanisms, e.g., upregulation of fibroblast growth factor (FGF)-23 production, to prevent excessive calcium uptake (for review, please see Wongdee et al. 2021 [8]). In other words, after several weeks of Ca+Vit D treatment, the action of calcium supplement was gradually diminished by the vitamin D-induced negative feedback regulation. However, as shown in Fig 6A, calcium and/or vitamin D plausibly promoted adult neurogenesis, which was sufficient to reduce anxiety-related behaviors and modulate locomotor activities in rats. Specifically, an increase in adult neurogenesis was able to alleviate anxiety and depression-related behaviors through hypothalamic-pituitary-adrenal axis in stressed or depressed rodents [26]. In addition, neurogenesis may also induce exploration behavior [73].

Regarding neuronal apoptosis in dentate gyrus, our TUNEL study indicated that supplementation of calcium chloride in drinking water was not toxic since it did not induce apoptosis of hippocampal neurons. Although certain regimens of calcium supplement, e.g., diet supplemented with 1.0% calcium carbonate (4 g/day; 8-week supplementation) vs. 0.2% calcium carbonate (normal diet), were reported to induce memory impairment in ICR mice as determined by Y-maze test and NOR [74], the present calcium chloride in drinking water *ad libitum* showed an increase in percent recognition index in rats (Fig 5C), suggesting that the outcome of calcium supplement was probably dependent on calcium compounds (calcium carbonate vs. calcium chloride), duration of treatment (4 vs. 8 weeks), animal species (mouse vs. rat) and/or mode of supplementation (calcium in diet vs. drinking water). Excessive calcium supplementation—particularly with hypercalcemia—is detrimental to neuronal activities. In contrast to the aforementioned beneficial effects of calcium and vitamin D supplements, calcium carbonate was reported to induce memory impairment by decreasing CREB expression, while excess vitamin D level also produced toxicity [74,75]. Moreover, an inappropriately high calcium and/or vitamin D intake can lead to kidney stone formation [76,77]. Therefore, a proper regimen of calcium/vitamin D supplementation should be further studied and optimized in order that it safely helps mitigate anxiety and enhance memory in osteoporotic patients with certain psychiatric diseases (e.g., mood disorder).

Regarding the limitations of the present study, the exact explanation why only vitamin D_3_-treated group showed anxiolytic-like behavior is unclear, but it might be due to the effect of calcium supplement that could reduce the expression of VDR [78], thereby obscuring the positive effect of vitamin D_3_. In addition, female rats were not investigated in the present study; therefore, further experiments are required to confirm the present findings in female rats, which may somewhat have sex difference. For example, the intestinal absorptive cells of female rats often express higher calcium transporter protein levels (e.g., TRPV6 calcium channel) than those of male rats, which might, in turn, affect the response of female rats to oral calcium supplement. Moreover, future determinations of BrdU incorporation and biomarkers of mature hippocampal neuron [e.g., hexaribonucleotide binding protein-3 (NeuN) and prospero homeobox protein 1 (Prox1) in dentate gyrus] in calcium/vitamin D_3_-supplemented rats are worth exploring.

## Conclusions

The 4-week calcium and/or vitamin D supplementation were demonstrated to help alleviate anxiety and enhance recognition memory in rats, as evaluated by EPM and NOR tests, respectively. Although the anxiolytic-like action of calcium supplement was not detected by EPM, it was able to enhance locomotor activity as determined by OFT. Both calcium and vitamin D also upregulated hippocampal DCX protein expression, which was an indirect proxy indicator of neurogenesis, while having no significant effect on hippocampal cell apoptosis. An increase in DCX expression in the hippocampus was probably a mechanism to help protect brain against anxiety and to improve the hippocampus-dependent cognitive function. Nevertheless, it is important to note that inappropriate calcium supplementation (e.g., prolonged high-dose calcium supplement) may induce certain adverse outcomes, such as an increased risk of dementia [19]. Therefore, our findings have provided foundation for further development of better and safe calcium/vitamin D supplement regimens that help improve mood and memory for individuals with osteopenia, osteoporosis or fracture, agreeing with SDG 3 (Good Health and Well-being) of United Nations Sustainable Development Goals (SDG).

## Supporting information

S1 File Original Western blot images of Fig 6A. Original Western blot images of protein expression levels of hippocampal doublecortin (DCX) and β-actin in rats supplemented with calcium and/or vitamin D_3_ for 4 weeks.
(PDF)

S2 File Research raw data of all experiments. The tables show raw data of body weight, daily food and water intakes; serum and urinary calcium and serum 25(OH)D_3_; parameters measured by elevated plus-maze (EPM), open field test (OFT), and novel object recognition (NOR); and doublecortin expression and TUNEL-positive cells in hippocampus in control, calcium chloride (Ca), vitamin D3 (Vit D), and (Ca+Vit D) group.
(PDF)
