# Supplementary figures and images for: Oral calcium and vitamin D supplements differentially alter exploratory, anxiety-like behaviors and memory in male rats
Source: PLoS One. 2023 Aug 11;18(8):e0290106. doi: 10.1371/journal.pone.0290106 (PMC10420380; doi:10.1371/journal.pone.0290106)

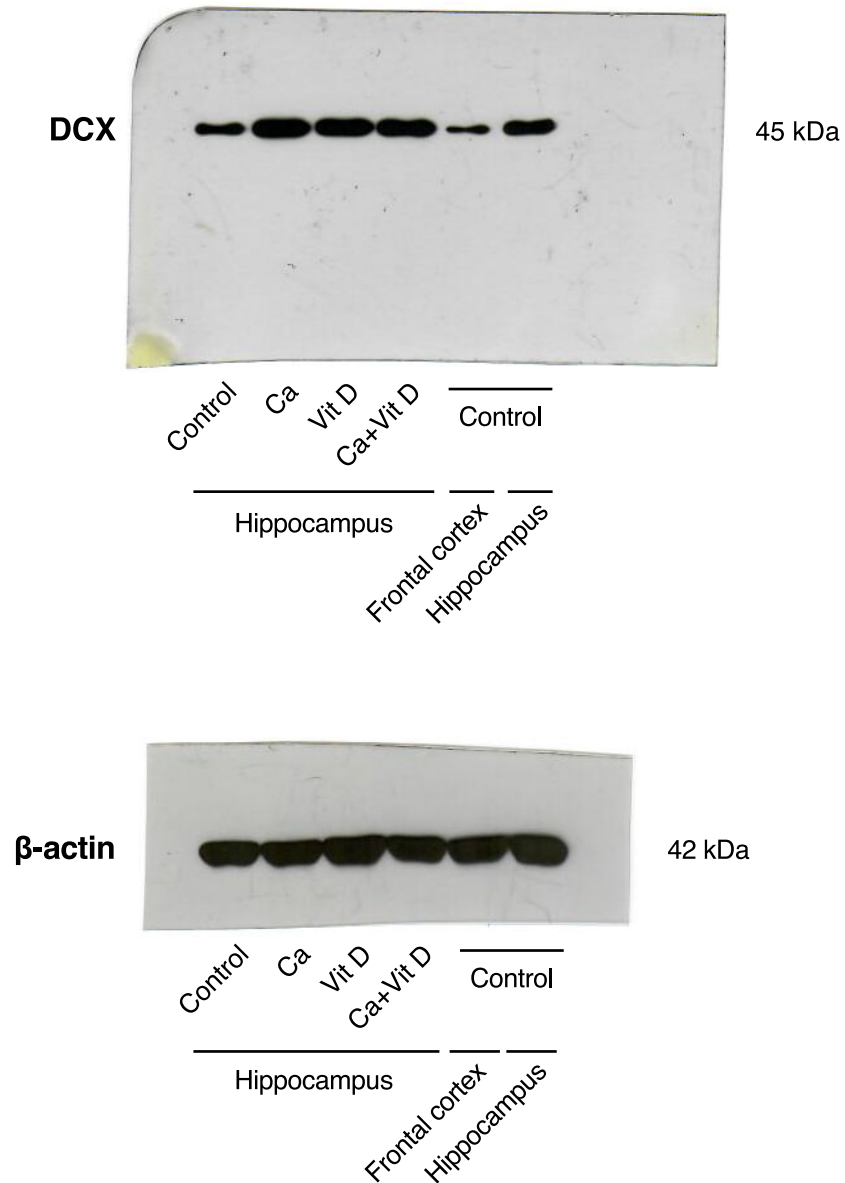

**Supplementary Figure S1:** Lapmanee et al.

Original blot images of Fig. 6A

Supplement: S1 File — Original Western blot images of protein expression levels of hippocampal doublecortin (DCX) and β-actin in rats supplemented with calcium and/or vitamin D3 for 4 weeks. (PDF) [file pone.0290106.s001.pdf]
