# Supplementary material for: Oral calcium and vitamin D supplements differentially alter exploratory, anxiety-like behaviors and memory in male rats
Source: PLoS One. 2023 Aug 11;18(8):e0290106. doi: 10.1371/journal.pone.0290106 (PMC10420380; doi:10.1371/journal.pone.0290106)
Supplement: S2 File — The tables show raw data of body weight, daily food and water intakes; serum and urinary calcium and serum 25(OH)D3; parameters measured by elevated plus-maze (EPM), open field test (OFT), and novel object recognition (NOR); and doublecortin expression and TUNEL-positive cells in hippocampus in control, calcium chloride (Ca), vitamin D3 (Vit D), and (Ca+Vit D) group. (PDF) [file pone.0290106.s002.pdf]

RESEARCH DATA

S1 Table. Starting and final body weight of individual rats in control, calcium chloride (Ca), vitamin D_3 (Vit D), and (Ca+Vit D) group

Body weight (kg)	No.	Control	Ca	Vit D	Ca+Vit D
**Starting BW**	1	201.70	205.40	206.80	207.10
	2	200.00	207.50	205.60	202.40
	3	206.50	198.80	213.20	198.00
	4	200.20	202.60	204.80	171.00
	5	198.80	230.00	230.00	220.00
	6	234.20	224.60	210.40	230.00
	7	224.50	209.90	200.00	203.70
	8	201.60	205.70	230.00	210.50
**Mean ± SEM**		**208.44 ± 4.72**	**210.56 ± 3.87**	**212.60 ± 4.04**	**205.34 ± 6.12**
**Final BW**	1	362.00	372.10	210.50	204.00
	2	340.80	382.10	220.00	277.00
	3	351.90	367.40	244.10	205.40
	4	370.80	344.20	189.80	282.50
	5	375.40	353.10	230.90	278.20
	6	400.00	397.30	212.50	153.50
	7	374.10	393.30	190.00	203.70
	8	381.10	376.10	314.00	210.50
**Mean ± SEM**		**369.52 ± 6.43**	**373.20 ± 6.48**	**226.48 ± 14.12**	**226.85 ± 16.59**

S2 Table. Food and water intakes of each rat in control, calcium chloride (Ca), vitamin D_3 (Vit D), and (Ca+Vit D) group

	No.	Control	Ca	Vit D	Ca+Vit D
**Food intake**	1	18.02	20.00	14.96	13.94
**(g/day)**	2	18.22	18.34	12.5	16.54
	3	18.58	18.9	16.52	12.62
	4	19.00	17.88	12.02	17.44
	5	19.48	19.34	13.52	13.76
	6	20.16	20.22	13.44	13.56
	7	18.96	19.36	16.34	13.40
	8	21.18	18.5	18.28	13.06
**Mean ± SEM**		**19.20 ± 0.37**	**19.07 ± 0.28**	**14.70 ± 0.77**	**14.29 ± 0.61**
**Drinking (mL/day)**	1	24.80	32.88	35.66	26.14
	2	34.92	29.70	33.88	25.48
	3	32.78	30.28	36.84	24.70
	4	28.64	33.76	39.10	34.70
	5	25.06	31.62	30.70	37.62
	6	28.18	29.80	34.68	40.28
	7	39.20	35.90	34.98	45.14
	8	25.26	31.62	58.92	44.52
**Mean ± SEM**		**19.86 ± 1.87**	**31.95 ± 0.76**	**38.10 ± 3.09**	**34.82 ± 2.99**

S3 Table. Serum and urinary calcium and serum 25(OH)D_3 of each rat in control, calcium chloride (Ca), vitamin D_3 (Vit D), and (Ca+Vit D) group

	No.	Control	Ca	Vit D	Ca+Vit D
**Serum calcium**	1	12.00	11.40	11.00	11.40
**(mg/dL)**	2	11.50	11.30	11.00	11.00
	3	11.60	11.70	10.20	12.80
	4	11.60	11.40	12.00	11.20
	5	11.60	13.30	12.10	10.60
	6	12.50	12.50	9.50	10.50
	7	11.30	12.00	10.20	12.00
	8	10.00	11.00	12.00	12.50
**Mean ± SEM**		**11.52 ± 0.25**	**11.83 ± 0.27**	**11.00 ± 0.35**	**11.50 ± 0.30**
**Urinary calcium**	1	22.50	47.80	15.3	22.00
**(mg/dL)**	2	22.60	34.90	20.00	19.80
	3	40.10	29.90	14.40	19.70
	4	33.90	51.40	34.80	25.70
	5	26.60	65.00	18.90	33.40
	6	30.00	30.00	20.00	20.00
	7	35.00	35.00	30.00	35.00
	8	20.00	40.00	25.00	30.00
**Mean ± SEM**		**28.84 ± 2.51**	**41.75 ± 4.32**	**22.30 ± 2.52**	**25.70 ± 2.24**
**Serum 25(OH)D_3**	1	63.70	52.60	81.20	195.60
**(ng/dL)**	2	50.00	54.40	117.00	87.60
	3	60.70	50.40	138.00	164.40
	4	48.60	61.60	138.00	103.00
	5	53.30	58.90	148.00	110.00
	6	60.00	50.00	90.00	115.00
	7	65.00	60.00	120.00	100.00
	8	75.00	70.00	100.00	95.00
**Mean ± SEM**		**59.54 ± 3.10**	**57.24 ± 2.39**	**116.53 ± 8.61**	**121.33 ± 13.47**

S4 Table. Raw data of the parameters measured by Elevated plus-maze (EPM) test in control, calcium chloride (Ca), vitamin D_3 (Vit D), and (Ca+Vit D) group

	No.	Control	Ca	Vit D	Ca+Vit D
**% Open arm time**	1	76.25	70.45	80.18	66.65
	2	76.85	81.12	93.34	70.42
	3	95.10	67.76	63.41	76.95
	4	82.39	51.16	71.85	74.00
	5	52.76	75.33	68.55	64.07
	6	37.49	28.28	82.75	80.01
	7	52.56	41.18	82.61	63.34
	8	38.88	37.79	73.48	67.41
**Mean ± SEM**		**64.04 ± 7.57**	**56.63 ± 6.93**	**77.02 ± 3.37**	**70.36 ± 2.16**
**% Open arm entry**	1	22.50	47.80	15.3	22.00
	2	22.60	34.90	20.00	19.80
	3	40.10	29.90	14.40	19.70
	4	33.90	51.40	34.80	25.70
	5	26.60	65.00	18.90	33.40
	6	30.00	30.00	20.00	20.00
	7	35.00	35.00	30.00	35.00
	8	20.00	40.00	25.00	30.00
**Mean ± SEM**		**54.57 ± 5.01**	**52.45 ± 4.42**	**66.69 ± 3.96**	**65.43 ± 2.29**
**Anxiety index**	1	0.35	0.33	0.27	0.35
	2	0.28	0.32	0.11	0.37
	3	0.12	0.38	0.37	0.23
	4	0.28	0.52	0.32	0.30
	5	0.52	0.26	0.35	0.37
	6	0.62	0.69	0.27	0.27
	7	0.52	0.56	0.19	0.37
	8	0.58	0.58	0.38	0.32
**Mean ± SEM**		**0.41 ± 0.06**	**0.46 ± 0.05**	**0.28 ± 0.03**	**0.32 ± 0.02**

S5 Table. Raw data of the parameters measured by Open field test (OFT) in control, calcium chloride (Ca), vitamin D_3 (Vit D), and (Ca+Vit D) group

	No.	Control	Ca	Vit D	Ca+Vit D
**Inner zone time (s)**	1	64.87	138.21	155.96	127.29
	2	138.90	160.52	103.50	55.53
	3	152.20	115.19	152.85	80.37
	4	205.21	112.41	132.17	149.42
	5	92.78	82.00	84.00	107.06
	6	92.68	69.68	99.46	122.22
	7	72.89	63.21	90.00	200.13
	8	159.89	102.14	102.69	148.37
**Mean ± SEM**		**122.3 ± 17.40**	**105.42 ± 11.86**	**115.08 ± 9.93**	**123.80 ± 15.77**
**Outer zone time (s)**	1	235.13	161.79	144.04	172.71
	2	161.10	139.48	196.5	244.47
	3	147.80	184.81	147.15	219.63
	4	94.79	187.59	167.83	150.58
	5	207.22	218.00	216.00	192.94
	6	207.32	230.32	200.54	177.78
	7	227.11	236.79	210.00	99.87
	8	140.11	197.86	197.31	151.63
**Mean ± SEM**		**177.57 ± 17.40**	**194.58 ± 11.86**	**184.92 ± 9.93**	**176.20 ± 15.77**
**Total lines crossed**	1	111	174	128	134
	2	215	160	125	207
	3	148	225	144	124
	4	195	269	199	165
	5	230	232	130	106
	6	174	211	113	225
	7	188	281	120	138
	8	200	232	200	200
**Mean ± SEM**		**182.63 ± 13.48**	**223.00 ± 14.74**	**144.88 ± 12.32**	**162.38 ± 15.46**

S5 Table. Raw data of the parameters detected by Open field test (OFT) in control, calcium chloride (Ca), vitamin D_3 (Vit D), and (Ca+Vit D) group (continue)

	No.	Control	Ca	Vit D	Ca+Vit D
**Rearing (time)**	1	40	40	21	35
	2	26	46	46	29
	3	40	52	37	32
	4	43	65	51	42
	5	41	55	49	25
	6	24	55	44	66
	7	30	51	20	62
	8	28	50	54	65
**Mean ± SEM**		**34.00 ± 2.73**	**51.75 ± 2.58**	**40.25 ± 4.67**	**44.50 ± 6.07**
**Grooming (time)**	1	2	2	2	2
	2	3	2	2	2
	3	1	1	1	2
	4	1	1	2	4
	5	3	2	3	3
	6	3	1	4	3
	7	2	2	0	3
	8	0	3	2	2
**Mean ± SEM**		**1.88 ± 0.40**	**1.75 ± 0.25**	**2.00 ± 0.42**	**2.63 ± 0.26**

S6 Table. Raw data of the parameters measured by Novel object recognition (NOR) in control, calcium chloride (Ca), vitamin D_3 (Vit D), and (Ca+Vit D) group

	No.	Control	Ca	Vit D	Ca+Vit D
**Total exploration time (s)**	1	41.02	58.14	77.3	103.58
	2	34.11	56.39	52.91	96.77
	3	122.76	121.29	88.31	33.76
	4	100.46	108.76	102.2	143.07
	5	81.37	44.32	74.15	74.79
	6	24.61	108.36	126.21	81.44
	7	56.43	90.51	58.92	60.78
	8	78.84	82.62	21.79	107.74
**Mean ± SEM**		**67.45 ± 12.13**	**83.80 ± 10.04**	**75.22 ± 11.28**	**87.74 ± 11.68**
**Recognition index (%)**	1	51.24	66.70	31.88	74.71
	2	30.46	79.11	24.40	46.51
	3	20.64	33.09	16.68	58.59
	4	32.59	28.54	58.85	40.41
	5	31.65	39.94	24.72	29.88
	6	22.31	67.08	31.07	53.86
	7	27.49	45.34	54.35	28.96
	8	26.10	41.75	39.51	49.69
**Mean ± SEM**		**30.31 ± 3.35**	**50.19 ± 6.49**	**35.18 ± 5.25**	**47.83 ± 5.37**

S7 Table. Data of doublecortin expression and TUNEL-positive cells in hippocampus in control, calcium chloride (Ca), vitamin D_3 (Vit D), and (Ca+Vit D) group

	**No.**	**Control**	**Ca**	**Vit D**	**Ca+Vit D**
**DCX protein (Normalized to β-actin)**	1	1.00	2.98	3.31	3.04
	2	1.00	3.11	1.05	1.68
	3	1.00	0.95	2.06	1.15
	4	1.00	1.87	2.40	0.73
	5	1.00	2.33	1.57	5.20
	6	1.00	1.89	3.82	2.10
	7	1.00	2.00	2.50	2.50
	8	1.00	2.50	3.50	2.50
**Mean ± SEM**		**1.00 ± 0.00**	**2.20 ± 0.25**	**2.52 ± 0.34**	**2.23 ± 0.49**
**TUNEL-positive cells (%)**	1	0.19	0.39	0.55	0.19
	2	1.58	3.07	4.78	2.31
	3	2.35	6.23	11.02	14.81
	4	6.71	13.71	15.49	15.22
**Mean ± SEM**		**2.71 ± 1.41**	**5.85 ± 2.88**	**7.96 ± 3.31**	**8.13 ± 4.00**
